# Retro-Iliac Ureter: A Case Report of an Unusual Cause of Mid-ureteric Obstruction

**DOI:** 10.7759/cureus.93525

**Published:** 2025-09-29

**Authors:** Souvik Chatterjee, Zochampuia Ralte, Debansu Sarkar

**Affiliations:** 1 Urology, Institute of Post Graduate Medical Education & Research, Kolkata, IND

**Keywords:** hydronephrosis, mayer-rokitansky-küster-hauser syndrome type ii, open modified gibson incision, rare congenital anomaly, retro-iliac ureter

## Abstract

Retro-iliac ureter, a rare congenital anomaly in which the ureter courses behind the iliac vessels, is infrequently reported in the urological literature. We describe the case of a 27-year-old woman with Mayer-Rokitansky-Küster-Hauser (MRKH) syndrome type II who presented with recurrent urinary tract infections (UTIs) and persistent right flank pain. Intraoperative retrograde pyelogram (RGP) demonstrated a markedly dilated right ureter with contrast hold-up near the sacroiliac joint. Surgical exploration confirmed a retro-iliac course of the ureter. The patient underwent successful anterior transposition and anastomosis of the ureter. This case illustrates the diagnostic challenges and surgical management of the retro-iliac ureter, particularly when associated with complex congenital anomalies such as MRKH syndrome. To our knowledge, this may be the first reported case of these two rare anomalies being combined.

## Introduction

Retro-iliac ureter is a congenital anomaly in which the ureter passes posterior to the iliac vessels. First identified by Rotter during autopsy in 1935 [1] and later described in clinical settings by Dees [2] and Corbus et al. [3], this condition remains exceedingly rare, with fewer than 30 cases reported worldwide. It is often discovered intraoperatively due to its subtle or ambiguous radiological features. Retro-iliac ureter typically results from vascular developmental anomalies during embryogenesis, particularly involving persistent primitive vascular structures. Given its nonspecific clinical presentation and imaging findings, accurate diagnosis often necessitates surgical exploration. This report highlights a unique case of retro-iliac ureter in a female with Mayer-Rokitansky-Küster-Hauser (MRKH) syndrome type II, a combination not previously documented in the literature to our knowledge.

## Case presentation

A 27-year-old female presented with a four-year history of recurrent urinary tract infections (UTIs) and persistent right lower abdominal and flank pain over the preceding year. Her past medical history included anal atresia, treated via posterior sagittal anorectoplasty (PSARP) and colostomy in infancy, with subsequent colostomy closure at two years of age. She was also a diagnosed case of Mayer-Rokitansky-Küster-Hauser (MRKH) syndrome type II, presenting with primary amenorrhea, infertility, a blind vaginal pouch, and hypothyroidism.

**Figure 1:**
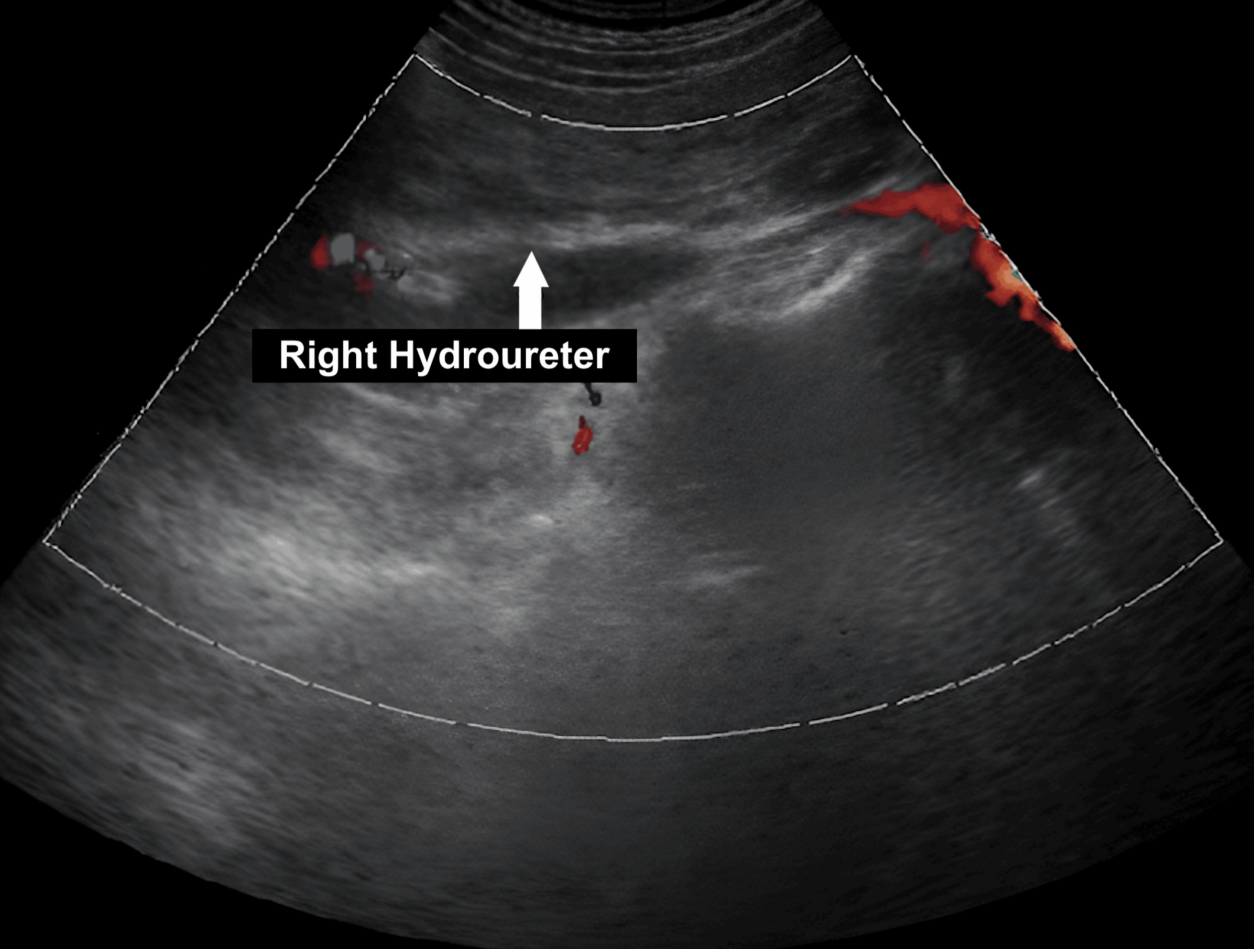
Right hydroureteronephrosis

The physical examination revealed right-sided lower abdominal and flank tenderness. The laboratory evaluation, including follicle-stimulating hormone (FSH) levels, was within normal limits. Ultrasonography showed right-sided hydroureteronephrosis and absence of the left kidney, uterus, and ovaries (Figure 1).

**Figure 2:**
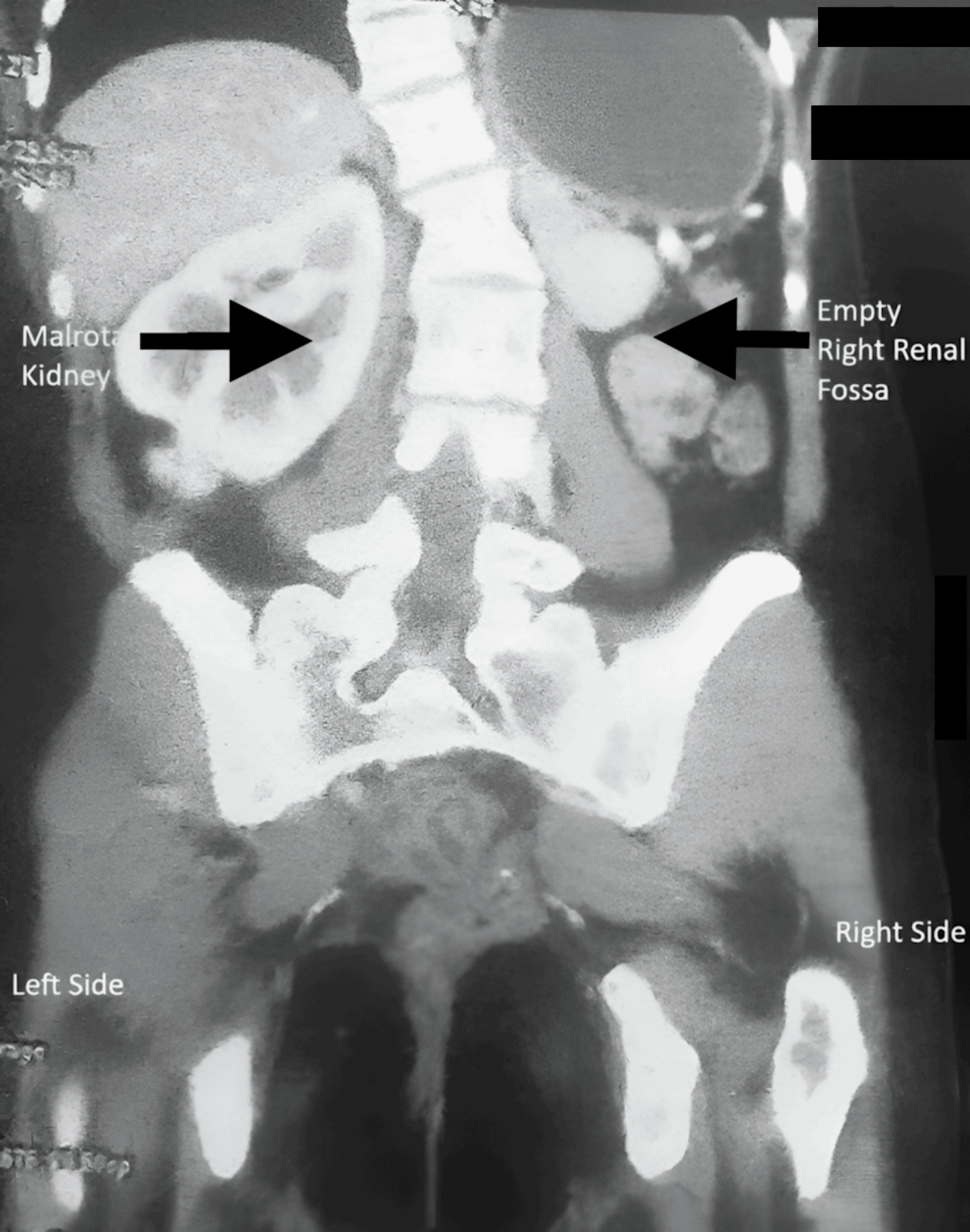
Anterior malrotation of the right kidney, absent left kidney

**Figure 3:**
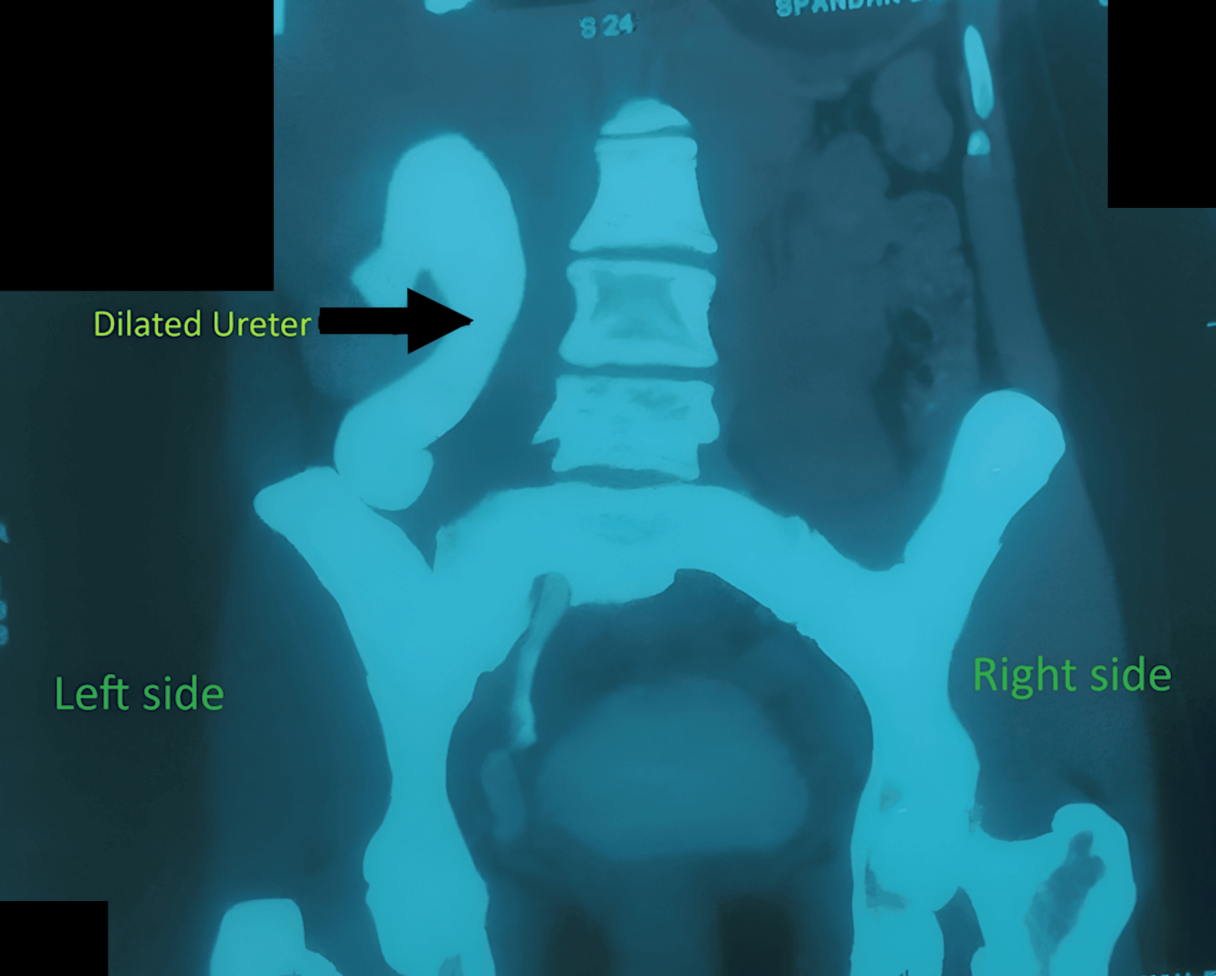
Dilated right ureter

Contrast-enhanced computed tomography (CECT) of the abdomen demonstrated anterior malrotation of the right renal pelvis (Figure 2) and proximal ureteral kinking with significant upstream dilatation (Figure 3).

**Figure 4:**
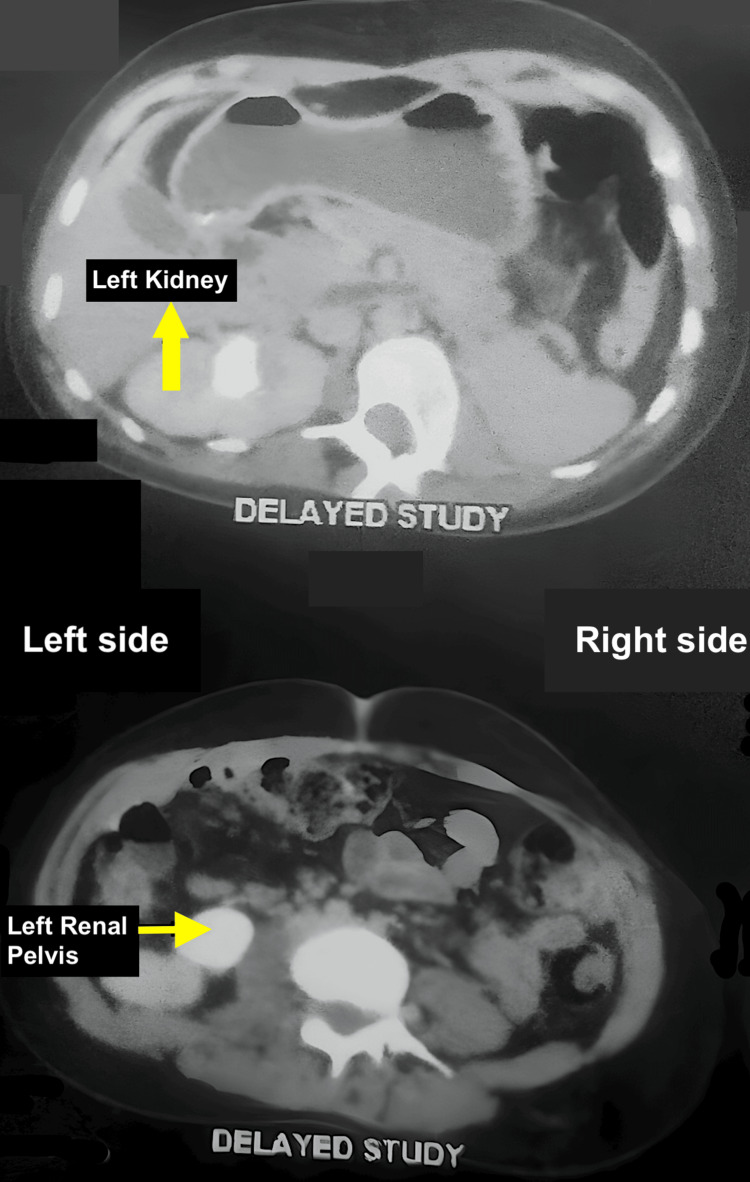
Right hydroureteronephrosis with absent left kidney

The left kidney and ureter were not visualized, suggesting agenesis (Figure 4).

**Figure 5:**
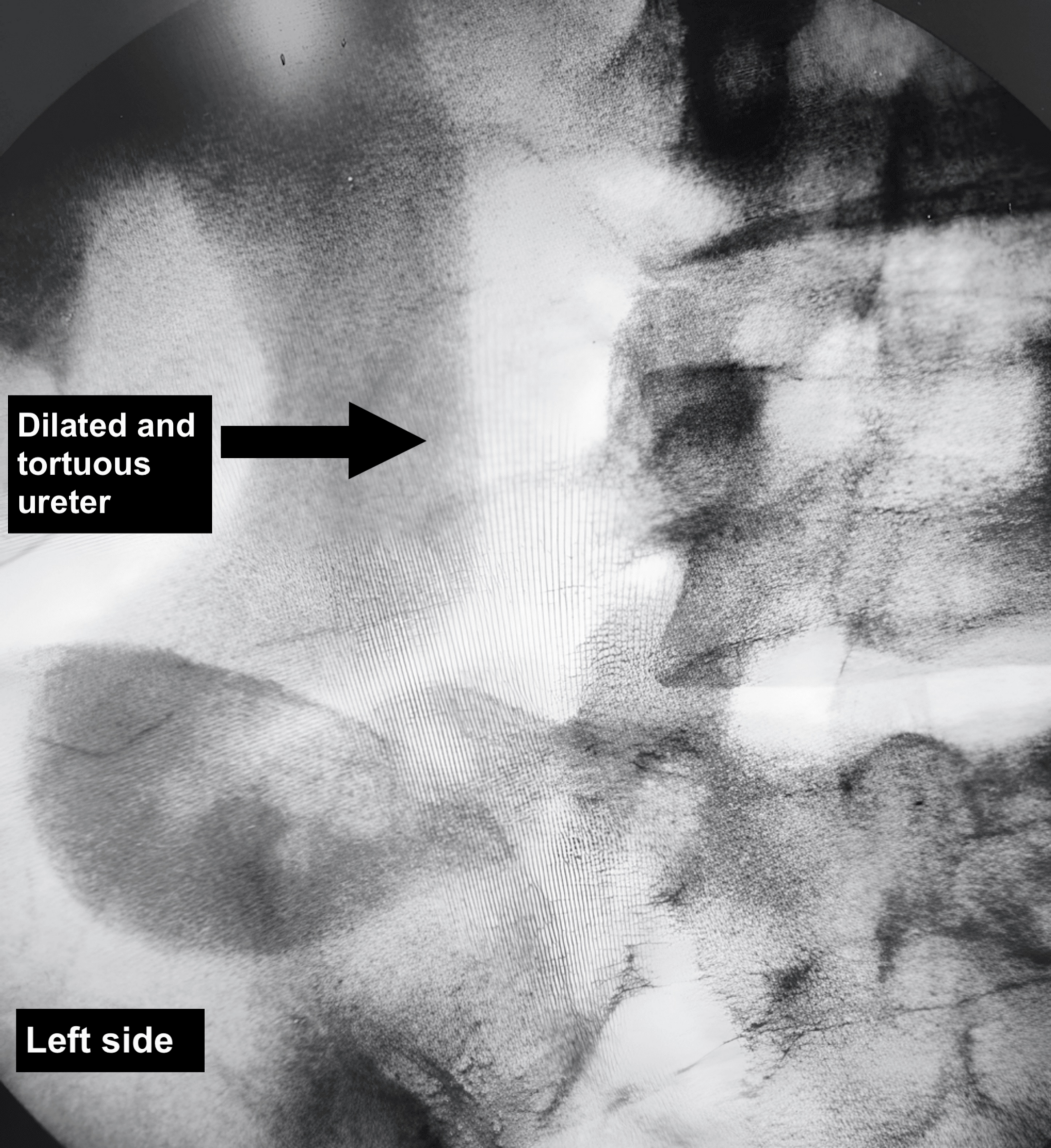
Intraoperative RGU showing dilated right ureter RGU: retrograde urethrogram

**Figure 6:**
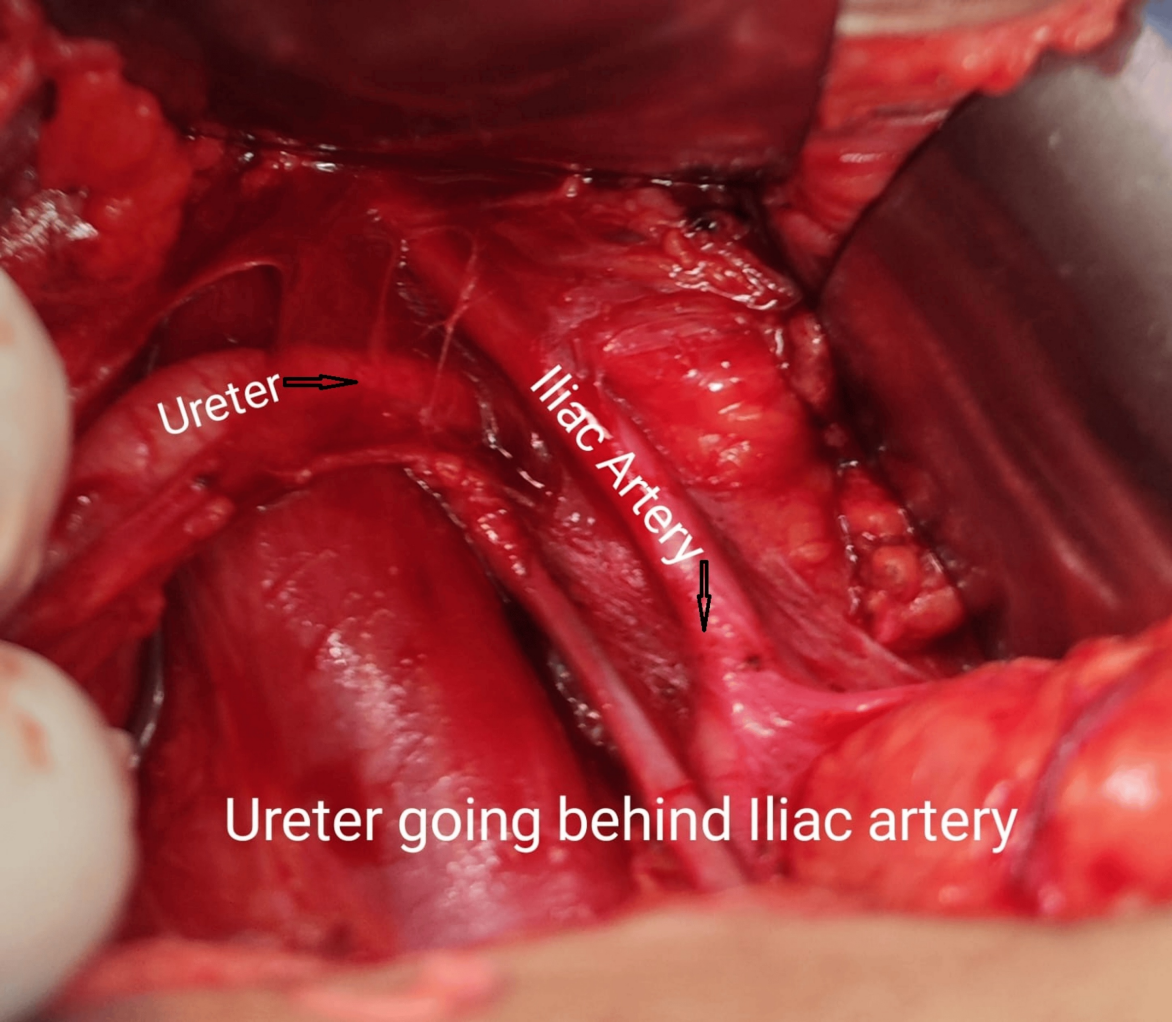
Intraoperative image showing the ureter passing behind the right common iliac artery

**Figure 7:**
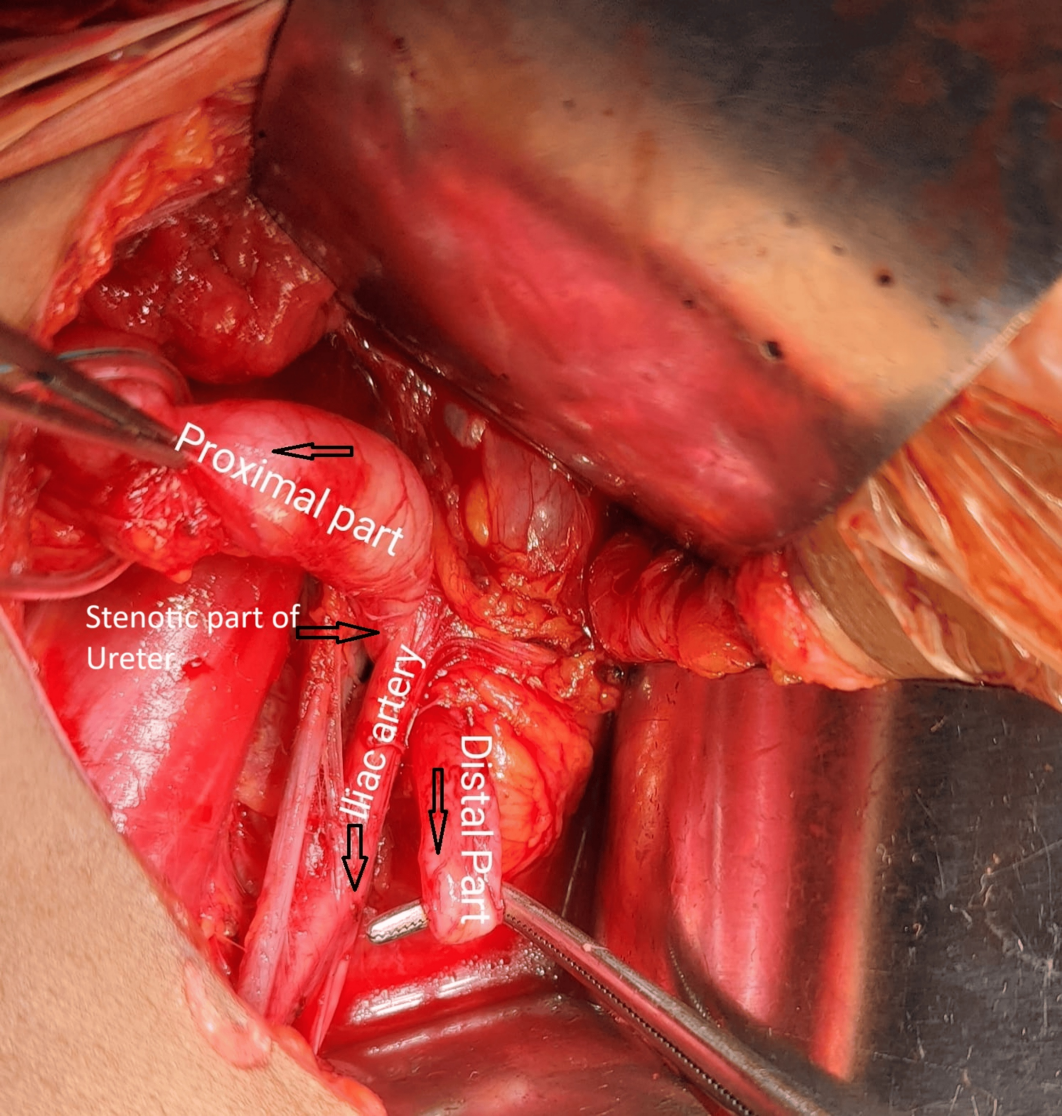
Intraoperative image showing the ureteric stenotic part posterior to the common iliac artery

Given these findings, surgical intervention was planned. Intraoperative retrograde pyelography (RGP) confirmed a grossly dilated and tortuous right ureter up to the level of the sacroiliac joint (Figure 5) with a 15-minute contrast hold-up. Through a modified Gibson incision, exploration revealed that the ureter was coursing posterior to the right common iliac artery (Figure 6), consistent with a retro-iliac course. A stenotic segment in the retro-iliac portion (Figure 7) was excised and submitted for histopathological evaluation. An end-to-end ureteroureterostomy was performed using 2-0 polyglactin suture over a 6/26 Fr double-J stent. The postoperative course was uneventful, and the patient was discharged on the seventh postoperative day.

Histopathological analysis of the excised ureteric segment revealed chronic inflammatory changes, accompanied by fibrosis and hyalinization, consistent with stricture formation.

## Discussion

Retro-iliac ureter is a rare congenital anomaly in which the ureter travels posterior to the iliac vessels, often leading to obstructive uropathy. The condition is challenging to diagnose preoperatively due to nonspecific radiologic findings and is frequently identified during surgery for presumed mid-ureteric obstruction [1-3]. Imaging may reveal hydroureteronephrosis extending to the L4-L5 level, which helps distinguish it from a retrocaval ureter, where the ureter is typically dilated above the L3 level [4].

The condition is believed to result from aberrant vascular development during embryogenesis. Normally, the dorsal branch of the umbilical artery gives rise to the iliac arteries, while the ventral root regresses. Failure of this regression and persistence of the ventral root can lead to the ureter being trapped behind the developing vessels, resulting in a retro-iliac course [4-6]. Gray and Skandalakis supported this embryologic mechanism, proposing that the failure of proper vascular remodeling is the principal cause [6].

Nguyen et al. reviewed 24 cases of retro-iliac ureter across a broad age range (3 to 70 years), with an equal male-to-female distribution and a predominance of right-sided anomalies. Common symptoms included recurrent UTIs, flank pain, lower abdominal discomfort, and hypertension. In some instances, diagnosis was incidental during unrelated surgical procedures or autopsies [4]. Management primarily involves ureteral transposition, ureteroureterostomy, or ureteroneocystostomy, depending on anatomical considerations. In advanced cases, nephrectomy may be warranted when renal function is severely compromised [7,8].

In the present case, the patient had MRKH syndrome type II, characterized by vaginal agenesis and renal anomalies, such as unilateral renal agenesis or ectopia in 40-60% of cases [9]. While genitourinary malformations are well-documented in MRKH, the coexistence of retro-iliac ureter with unilateral renal agenesis in such a patient has not been previously reported to our knowledge, making this case particularly notable. The condition likely arose from concomitant disruptions in Müllerian and mesonephric duct development [9,10].

Accurate diagnosis of retro-iliac ureter remains elusive on imaging alone. Findings such as ureteral kinking, sacroiliac-level obstruction, or ureteral tortuosity should raise suspicion. Intraoperative findings continue to be the gold standard for confirming the diagnosis and guiding definitive management [4,5].

## Conclusions

Retro-iliac ureter should be considered in the differential diagnosis of unexplained mid-ureteric obstruction, particularly when imaging reveals proximal dilatation near the iliac vessels. While rare, its association with complex congenital syndromes such as MRKH type II adds to the diagnostic challenge. Surgical exploration remains essential for definitive diagnosis and management. Our report underscores the importance of considering vascular anomalies in the evaluation of ureteric obstruction, especially in patients with associated congenital malformations.
